# Early discontinuation of combination antibiotic therapy in severe community-acquired pneumonia: a retrospective cohort study

**DOI:** 10.1186/s12879-023-08493-5

**Published:** 2023-09-18

**Authors:** Pauline Guillot, Flora Delamaire, Arnaud Gacouin, Benoit Painvin, Caroline Piau, Florian Reizine, Mathieu Lesouhaitier, Jean-Marc Tadié, Adel Maamar

**Affiliations:** 1 CHU Rennes, Service de Maladies Infectieuses Et Réanimation Médicale, Hôpital Pontchaillou, Université de Rennes 1, 2, Rue Henri Le Guilloux, 35033 Rennes Cedex 9, France; 2 Faculté de Médecine, Université de Rennes 1, Unité INSERM CIC 1414, IFR 140, Rennes, France; 3 CHU Rennes, Service de Bactériologie, Hôpital Pontchaillou, Université de Rennes 1, 2, Rue Henri Le Guilloux, 35033 Rennes Cedex 9, France

**Keywords:** Community-acquired pneumonia, Legionella, Antibiotic stewardship, Critically ill patients

## Abstract

**Background:**

Severe community-acquired pneumonia (SCAP) is commonly treated with an empiric combination therapy, including a macrolide, or a quinolone and a β-lactam. However, the risk of *Legionella* pneumonia may lead to a prolonged combination therapy even after negative urinary antigen tests (UAT).

**Methods:**

We conducted a retrospective cohort study in a French intensive care unit (ICU) over 6 years and included all the patients admitted with documented SCAP. All patients received an empirical combination therapy with a β-lactam plus a macrolide or quinolone, and a *Legionella* UAT was performed. Macrolide or quinolone were discontinued when the UAT was confirmed negative. We examined the clinical and epidemiological features of SCAP and analysed the independent factors associated with ICU mortality.

**Results:**

Among the 856 patients with documented SCAP, 26 patients had atypical pneumonia: 18 *Legionella pneumophila* (LP) serogroup 1, 3 *Mycoplasma pneumonia* (MP), and 5 *Chlamydia psittaci* (CP). UAT diagnosed 16 (89%) *Legionella* pneumonia and PCR confirmed the diagnosis for the other atypical pneumonia. No atypical pneumonia was found by culture only. Type of pathogen was not associated with a higher ICU mortality in the multivariate analysis.

**Conclusion:**

*Legionella pneumophila* UAT proved to be highly effective in detecting the majority of cases, with only a negligible percentage of patients being missed, but is not sufficient to diagnose atypical pneumonia, and culture did not provide any supplementary information. These results suggest that the discontinuation of macrolides or quinolones may be a safe option when *Legionella* UAT is negative in countries with a low incidence of *Legionella pneumonia*.

**Supplementary Information:**

The online version contains supplementary material available at 10.1186/s12879-023-08493-5.

## Introduction

Severe community-acquired pneumonia (SCAP) is a serious condition that poses a significant threat to public health. It is one of the leading causes of infectious mortality worldwide and a common reason for intensive care unit (ICU) admission [1, 2], particularly among immunocompromised and elderly patients with comorbidities. Despite recent advances in medical care, the incidence of SCAP continues to rise globally, and microbiological documentation is not obtained in many cases [2, 3].

Atypical pathogens, such as *Legionella pneumophila*, *Mycoplasma pneumoniae*, *Chlamydophila pneumoniae*, and *Chlamydia psittaci*, are a group of bacteria involved in SCAP, making it difficult to differentiate typical from atypical pneumonia based solely on clinical and biological features [4]. Moreover, 20% of *Legionella* pneumonia are admitted in ICU, with a high morbidity and mortality [5], but there is a wide heterogeneity between countries.

A wide range of pathogens can cause CAP, with overlapping clinical and biologic features making it difficult to clearly differentiate typical from atypical pneumonia [6]. Thus, the rapid identification of the responsible pathogen is crucial for the early initiation of appropriate treatment, which has been shown to significantly reduce mortality rates, especially in patients with bacteriemic pneumococcal pneumonia, *Legionella* pneumonia or septic shock [7]. Rapid diagnostic tests including urine antigen test (UAT), syndromic rapid multiplex polymerase chain-reaction (PCR) or specific PCR can help to quickly identify the responsible pathogen and initiate a specific treatment. In Europe, more than 90% of diagnoses of *Legionella* pneumonia are made by *Legionella* UAT [8], which is a highly effective diagnostic test. European and American guidelines recommend its use in patients admitted to ICU for SCAP [9, 10]. So far, the recommended empirical antibiotic treatment for SCAP is an association of a β-lactam plus a macrolide or a respiratory fluoroquinolone [9], but adherence to these guidelines is still insufficient [11]. Furthermore, the strategy for de-escalation or discontinuation of antibiotic treatments in critically ill patients is still debated, with specific data being scarce, especially regards to the region incidence of *Legionella* pneumonia.

The present study hypothesizes that the early interruption of macrolide or quinolone after a negative *Legionella* UAT is safe in critically ill patients admitted in a low incidence region of *Legionella* pneumonia. For that purpose, we retrospectively analysed all the consecutive documented SCAP admitted in our centre.

## Materials and methods

### Patients and setting

We conducted a retrospective monocentre cohort study in a medical ICU in a French teaching hospital. We included all consecutive patients aged over 18 years admitted for a SCAP over a 6-year period, from January 1st, 2015, to December 31st, 2020. Patients with documented SCAP were identified through our computerized database.

This study has been performed in accordance with the Declaration of Helsinki and was approved by the Rennes University hospital’s ethical committee. Due to its retrospective nature, the informed consent was waived by the Rennes University hospital’s ethic committee (n° 21.165).

### CAP definition and severity

Diagnosis of CAP was consistent with published guidelines [10]. We defined a CAP as the association between clinical features of pneumonia (e.g., fever, cough, sputum production, and pleuritic chest pain), supported by lung imagery, usually chest radiography or computerized tomography scan, and a positive microbiological identification. We excluded all patients with no microbiological documentation. We separated the pneumonia in 2 groups in regards to the need for a macrolide or a respiratory fluoroquinolone. Thus, we considered an “atypical” pneumonia as a CAP due to *Legionella pneumophila*, *Mycoplasma pneumoniae*, *Chlamydophila pneumoniae*, or *Chlamydia psittaci* [9]. All other pathogens were categorized as “other” pneumonia.

Severity was defined as the need for ICU admission, usually severe hypoxemia, need for mechanical ventilation, hypotension requiring vasopressors, alteration of consciousness, or associated comorbidities.

### Microbiological identification

In all patients, *Legionella* and *Streptococcus pneumoniae* UAT were performed, and blood culture samples were collected in the hospital before the first dose of antibiotics. In addition, at least one respiratory sample was obtained in all patients before and/or immediately after intubation. Detection of influenza virus and SARS-CoV-2 used real-time reverse transcriptase PCR (RT-PCR) in respiratory specimen collected at the time of admission among patients admitted with respiratory failure.

Positive cultures were established when etiologic agents were identified from the blood, sputum, endotracheal aspiration, or bronchoalveolar lavage fluid. We also considered positive tests, such as specific UAT for *Legionella* or *Streptococcus pneumoniae*, PCR for *Legionella pneumophila*, *Mycoplasma pneumoniae*, *Chlamydophila pneumoniae*,*Chlamydia psittaci*, and RT-PCR for respiratory viruses. Specific PCR for atypical pneumonia agents were systematically performed in case of bilateral infiltrates and negative UAT. The RT-PCR assay detects influenza viruses A and B, adenovirus, bocavirus, coronavirus 229E/NL63, coronavirus OC43/HKU1, enterovirus, human metapneumovirus, and parainfluenza viruses 1 to 4.

### SCAP management

All patients were managed accordingly to published guidelines [10]. In our unit, empirical antibiotic regimen consisted in a combination therapy with a β-lactam (third-generation cephalosporin) effective against *Streptococcus pneumoniae* plus a macrolide (usually clarithromycin). Antibiotic regimen was secondarily adapted once the etiologic agent was identified. Macrolides were discontinued as soon as the *Legionella* UAT was negative.

### Data collection

The parameters were extracted from medical records through a standardized questionnaire. Data collected for all patients were as follows: age, gender, body mass index (BMI), history of previous diseases including chronic cardiovascular disease, chronic respiratory insufficiency, chronic obstructive pulmonary disease (COPD) according to the American Thoracic Society criteria [12], proven cirrhosis, pre-existing renal insufficiency, malignant disorders, immunocompromising condition such as corticosteroids and chemotherapy, smoking and alcohol at-risk drinking. Severity at admission was assessed through the simplified acute physiology score II (SAPS II) within the first 24 h and the sequential organ failure assessment score (SOFA) at admission. We also recorded the initial presentation including clinical features (fever, confusion, diarrhoea, myalgia, and arthralgia), common biological markers (natremia and liver enzymes) and the notion of a recent trip defined by an incubation period of two to ten days. Additionally, we recorded need and duration of mechanical ventilation, need for extracorporeal membrane oxygenation (ECMO), length of ICU and hospital stay, and mortality in ICU and at 28 days.

### Statistical analysis

Normally distributed continuous variables are presented as the means (standard deviations), whereas non-normally distributed data are presented as medians (interquartile ranges). Categorical variables are presented as numbers (percentages). For between groups comparison, Mann–Whitney test for continuous variables and a χ2 or Fisher’s exact test when more appropriate for categorical variables were used. A descendant stepwise logistic regression analysis was performed to identify variables independently associated with ICU mortality. Variables with a *p* value ≤ 0.20 in the univariate analysis were entered in the model, and results were expressed as odds ratios (OR) with their 95% confident interval (CI). Among related factors (SAPS II at admission and SOFA at intubation) only the most clinically relevant (SAPS II for severity) were included in the multivariate analysis model to minimize the effect of collinearity. Statistical analyses were performed using R 4.1.1 (R Foundation for Statistical Computing, Vienna, Austria), and p-values of less than 0.05 were considered significant.

## Results

From January 1st, 2015, to December 31st, 2020, 6068 patients were admitted in our ICU. One thousand three hundred and thirteen patients were admitted for a suspected SCAP. Among them, 457 were excluded due to the absence of microbiological documentation. Thus, we included 856 patients with a documented SCAP, 26 (3%) with atypical SCAP and 830 (97%) with other SCAP.

Characteristics of the patients with atypical SCAP are described in Table 1. Of note, 19 (73%) were male, and the median age was 53 [45–64] years. Fever was the main symptom (21/26, 81%), followed by diarrhoea and myalgia. The notion of recent travel was found for 10 patients (39%). Hyponatremia and hepatic cytolysis were present in 11 (42%) and 14 (54%) patients respectively. There was no significant difference in demographic characteristics, initial severity, and outcomes between atypical and other SCAP in univariate analysis (Table 2).

**Table 1:** Characteristics of patients with SCAP at ICU admission

	Atypical SCAP *n* = 26	Other SCAP *n* = 830
Demographics		
Age	53 [45–64]	62 [51–71]
Male gender	19 (73)	545 (66)
BMI	27 [24–34]	25 [21–29]
Comorbidities		
Heart disease	5 (19)	174 (21)
Diabetes	6 (23)	183 (22)
COPD	2 (8)	166 (20)
Chronic respiratory failure	0	18 (2)
Smoking	9 (35)	324 (39)
Alcohol abuse	4 (15)	116 (14)
Cirrhosis	0	81 (10)
Chronic kidney disease	3 (12)	108 (13)
Cancer	0	33 (4)
Hemopathy	1 (4)	92 (11)
Corticosteroid therapy	3 (12)	191 (23)
Chemotherapy	0	66 (8)
Others immunosuppressive drugs	2 (8)	31 (4)
Clinical presentation		
Fever	21 (81)	739 (89)
Confusion	5 (19)	133 (14)
Diarrhoea	12 (46)	81 (10)
Myalgia	8 (31)	183 (22)
Arthralgia	0	32 (4)
Travel	10 (39)	100 (12)
Biological presentation		
Natremia < 130 mmol/L	11 (42)	76 (9)
Hepatic cytolysis	14 (54)	207 (25)
ASAT > 100 U/L	13 (50)	173 (21)
ALAT > 100 U/L	8 (32)	118 (14)
Severity of disease		
SAPS II score	36 [25–48]	40 [27–58]
SOFA score	4 [2–7]	6 [4–9]
Vasopressors > 0.1 µg/kg/min	8 (31)	461 (56)

Data are presented as number (%) or median [interquartile range]

*Abbreviations*: *SCAP* severe community-acquired pneumonia, *BMI* body mass index, *COPD* chronic obstructive pulmonary disease, *ASAT* aspartate aminotransferase, *ALAT* alanine aminotransferase, *SAPS* simplified acute physiology score, *SOFA* sequential organ failure assessment

**Table 2:** Comparison of atypical SCAP and typical SCAP

	Atypical SCAP (*n* = 26)	Typical SCAP (*n* = 830)	*p* value
Age	53 [45–64]	62 [51–71]	0.05
Male gender	19 (73)	545 (66)	0.57
SAPS II score	36 [25–48]	40 [27–58]	0.26
Mechanical ventilation	15 (58)	537 (65)	0.6
Duration of ventilation	11 [7–17]	4 [0–12]	0.01
Length of stay in ICU	8 [4–14]	7 [3–15]	0.65
Mortality in ICU	4 (15)	151 (18)	0.91

Data are presented as number (%) or median [interquartile range]

**Fig. 1:**
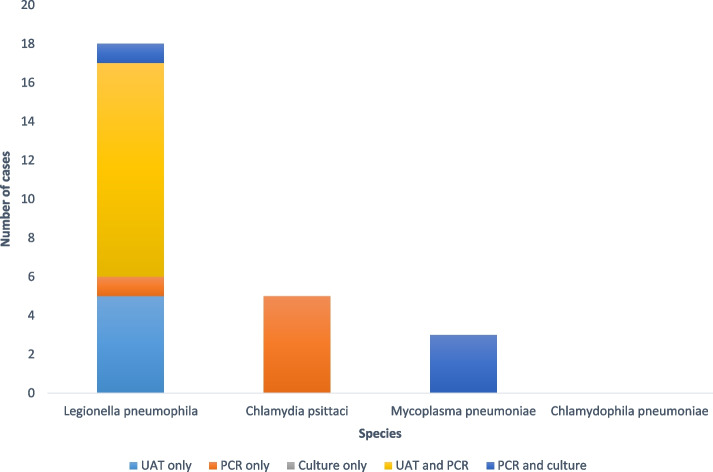
The histogram shows the number of positive cases of atypical severe community-acquired pneumonia (SCAP) by species and diagnostic technique. The bars are color-coded to represent the different techniques used: light blue for urinary antigen test (UAT) only, orange for polymerase chain reaction (PCR), grey for culture only, and dark blue for PCR and culture. The x-axis shows the different species of atypical bacteria, and the y-axis shows the number of positive cases

Microbiologic features are shown in Table 3. Of the 26 atypical pneumonia, 18 were due to *Legionella pneumophila* serogroup 1, 3 to *Mycoplasma pneumoniae*, and 5 to *Chlamydia psittaci*. There was no other *Legionella* serogroup or specie, no *Chlamydophila pneumonia,* and no co-infection. Among these patients, diagnoses were established as follows (Fig. 1, Supplementary Table 1): *Legionella pneumophila* serogroup 1 was diagnosed with a positive UAT in 16 patients (89%), and confirmed by a PCR in 11 patients. Diagnosis was established with a positive PCR in the two other patients, and one of them had a positive culture. *Mycoplasma pneumonia* was diagnosed with PCR and confirmed in a second respiratory sample for the 3 patients; *Chlamydia psittaci* was diagnosed in a single PCR for the 5 patients*.* No atypical pneumonia was diagnosed by culture alone.

**Table 3:** Microbiologic features

	Atypical SCAP *n* = 26	Other SCAP *n* = 1028
Bacteria	26 (100)	555 (54)
*Streptococcus pneumoniae*		210 (20)
*Staphylococcus aureus*		76 (7)
*Haemophilus influenza*		68 (7)
Enterobacteriaceae		55 (5)
Nonfermentive gram-negative bacteria		36 (4)
*Legionella pneumophila* serogroup 1	18 (69)	
Other *Legionella* spp	0	
*Mycoplasma pneumoniae*	3 (12)	
*Chlamydophila pneumoniae*	0	
*Chlamydia psittaci*	5 (19)	
Others		110 (11)
Viruses		379 (37)
SARS-CoV-2		169 (16)
Influenza		161 (16)
Others		49 (5)
Fungi and parasites		94 (9)
*Pneumocystis jirovecii*		70 (7)
*Aspergillus* spp		22 (2)
Others		2 (0)
Co-infections	0	201 (24)

Data are presented as number (%). Abbreviations: SCAP: severe community-acquired pneumonia

Macrolides were stopped for 10/856 (1.2%) patients based on a negative *Legionella* UAT (Supplementary Table 1). Among them, the final diagnoses were *Legionella* pneumonia for two patients, *Mycoplasma* pneumonia for 3 patients and *Chlamydia psittaci* for the 5 other patients. Specific therapy was reintroduced within the 48 first hours for all the patients.

Among the 4 patients who died in the ICU, 3 had a *Legionella* pneumonia and a positive UAT, while one patient had a *Chlamydia psittaci* infection diagnosed using specific PCR and had a negative *Legionella* UAT. The therapy directed against atypical bacteria was discontinued only for the patient with a negative UAT and subsequently reintroduced after the positive result of the *Chlamydia psittaci* PCR.

Duration of ventilation was statistically significantly longer for atypical SCAP, although need for mechanical ventilation was identical. Furthermore, two patients needed veno-venous ECMO and one had veno-arterial extracorporeal life support. However, length of stay and mortality in ICU were not significantly different between the 2 groups (Table 2). The multivariate analysis comparing survivors and non-survivors identified SAPS II score (OR = 1.04 by 1-point increment; 95% CI 1.03–1.05, *p* < 0.001) and the need for mechanical ventilation (OR = 5.4; 95% CI 2.5–13.3, *p* < 0.001) as independent factors associated with a higher mortality (Table 4).

**Table 4:** Univariate and multivariate analyses between survivors and non-survivors

	Univariate analysis			Multivariate analysis	
	Survivors	Non-survivors	*p* value	Odd Ratio [95% CI]	*p* value
Age	61 [50–70]	66 [57–74]	< 0.001		
Male gender	466 (67)	98 (63)	0.5		
SAPS II score	36 [24–52]	63 [47–76]	< 0.001	1.04 [1.03–1.05]	< 0.001
Atypical pneumonia	679 (97)	151 (97)	0.91		
Mechanical ventilation	404 (58)	148 (96)	< 0.001	5.4 [2.5–13.3]	< 0.001
Duration of ventilation	4 [0–11]	6 [2–18]	< 0.001		
Length of stay in ICU	7 [3–14]	7 [3–19]	0.43		

Data are presented as number (%) or median [interquartile range]

## Discussion

In this single centre study over a 6-years period, among 856 patients, only 26 patients (3%) were admitted for atypical SCAP. *Legionella pneumophila* serogroup 1 was the most common pathogen found and almost every time diagnosed by UAT. Among the atypical SCAP, there was no case diagnosed solely by the culture. Although the duration of mechanical ventilation was longer for atypical SCAP, we did not identify significantly different outcomes when compared with typical SCAP.

Atypical SCAP were relatively uncommon, accounting for only 3% of documented SCAP in our ICU, and *Legionella pneumophila* concerned 69% of cases. Similar prevalence of atypical SCAP were observed in Europe and France [10]. Consistently, we found only *Legionella pneumophila* serogroup 1 in our study, which is predominant in ICU because of its virulence [13, 14]. *Chlamydia psittaci* infection is the second pathogen involved in atypical SCAP in our study, in less than 1% of cases. It occurs in a particular context and history, where contact with birds is very frequently found [6]. Other pathogens involved in atypical pneumonia are even more uncommon and anecdotal. In particular, *Mycoplasma pneumoniae* appears to be rarely found in ICU patients compared to patients admitted in medical ward [15].

Most of the *Legionella* pneumonia were diagnosed by UAT in our study. This diagnostic test is the most widely performed in Europe and in USA, used in more than 90% of the cases [8]. Reasons are the ease of sampling and analysis, the rapidity of result, and the relatively low cost. Moreover, *Legionella* UAT sensitivity seems to be more important in ICU. Sensitivity varies according to serotype, exposure, and disease severity. Several studies showed a better sensitivity for serogroup 1 rather than other serogroups [16]. Moreover, sensibility increases with the severity of the infection, achieving 88% to 100%, probably due to an increased antigen urinary secretion in the most severe patients [17, 18]. For all these reasons, *Legionella* UAT seems to be a good diagnostic tool for *Legionella* pneumonia but is not a sufficient diagnostic tool to diagnose atypical pneumonia. In our study, macrolides or quinolones were wrongfully discontinued in only 10/856 (1.2%) patients. Therefore, in a low atypical pneumonia setting, it could be used to stop macrolide or quinolones in patients with SCAP.

It is worth noting that in our study, the majority of the diagnoses of atypical pneumonia were established using molecular techniques such as UAT or PCR, with only a few cases being culture-positive. In fact, none of the atypical pneumonia cases in our study were diagnosed based on culture alone, underscoring the limited utility of relying solely on culture in diagnosing these types of infections. Our findings suggest that waiting for culture results may not be necessary before considering discontinuing combination therapy in patients with a negative *Legionella* UAT.

Nevertheless, our findings highlight that although UAT could be sufficient to stop macrolides and quinolones because atypical pneumonia is uncommon, it is not sufficient for the diagnosis of atypical pneumonia and could benefit from the addition of PCR, as pointed out in the ATS/IDSA guidelines [9]. Thus, the clinicians should probably wait for the result of nucleic acid amplification testing before considering a de-escalation.

We found that initial severity was similar between atypical and typical SCAP groups. Although four of the 26 patients with atypical SCAP were de-escalated within the 24 first hours after admission in ICU, there was no significant difference in mechanical ventilation rate and ICU mortality between the two groups. Among them, only one patient had a *Legionella* pneumonia. This finding could probably be explained by the lower severity of non-*Legionella* atypical pneumonia, by an early first dose of macrolide or quinolone and a rapid reintroduction of a specific therapy (either macrolide, quinolone or doxycycline). While duration of mechanical ventilation was significantly longer in atypical SCAP patients, it did not influence ICU length of stay. A French study described 104 ICU patients admitted for atypical SCAP due to *Mycoplasma pneumoniae* and *Chlamydophila pneumonia*e. Although first-line antibiotics were ineffective in 40% of cases, mortality rate was similar to our study [11]. In addition, we did not find, in our multivariate analysis, independent factors associated with ICU survival others than those expected. Interestingly, the type of pneumonia was not associated with a different mortality rate. These findings suggest that the early discontinuation of combination therapy is not associated with an increased mortality in patients admitted for an atypical SCAP.

Along these lines, early discontinuation of macrolides has economic and bacterial ecology benefits. The estimated cost of a day's treatment with clarithromycin or spiramycin is estimated to approximately 15 euros. In addition, macrolides have an impact on the respiratory and intestinal microbiota [19, 20]. It has been shown that a 3-days antibiotic regimen of azithromycin or 7 days of clarithromycin increases the proportion of macrolide-resistant streptococci in the oral flora, which persist for at least 6 months [21]. However, the impact on the resistance after a short macrolide course appears to be limited. A prospective study of 48 critically ill patients did not find an increase in phenotypic or genotypic resistance for extended-spectrum β-lactamase Gram-negative bacteria, methicillin-resistant Staphylococcus aureus or vancomycin-resistant Enterococcus [22]. In our cohort, macrolides were systematically prescribed as a first line antibiotic, but stopped as soon as the *Legionella* UAT was obtained and negative. Thereby, the overwhelming majority of our patients received only one dose of macrolide.

For all these reasons, we believe that the early discontinuation of a combination therapy after a negative *Legionella* UAT is probably safe, as long as the history does not reveal contact with birds. This approach could help to reduce the global antibiotic consumption in regions with low *Legionella* pneumonia prevalence.

Some limitations should be acknowledged. First, as mentioned before, our study was retrospective; this design was required due to the low incidence of atypical SCAP in our region. Thus, our findings might not be generalized to other regions. Second, because we wanted to focus on atypical pneumonia, we voluntarily considered as typical all pneumonia not caused by atypical pathogens. Thereby, our analysis did not consider the impact of antibiotics in typical SCAP caused by non-bacterial pathogens, and the large variety of pathogens found in typical SCAP may affect the results. Third, our study focused on microbiologically documented SCAP and we did not assess the safety of early antibiotic discontinuation for non-documented pneumonia.

## Conclusion

In this 6-year retrospective single-centre cohort study, we found that only a small percentage of SCAP patients were admitted to the ICU due to atypical pneumonia. Furthermore, the *Legionella pneumophila* UAT proved to be highly effective in detecting the majority of cases, with only a negligible percentage of patients being missed, and culture did not provide any supplementary information, but is not sufficient to diagnose atypical pneumonia. Importantly, our results also suggest that the discontinuation of macrolides or quinolones may be a safe option when *Legionella* UAT is negative in countries with a low incidence of *Legionella pneumonia*, and that waiting for culture may not be necessary. This approach could lead to a reduction in antibiotic consumption, as well as positive economic and ecologic impacts.

### Supplementary Information

**Additional file 1: Supplementary Table 1.** Diagnostic methods and treatment at admission of patients with atypical SCAP.

## Data Availability

The data that support the findings of this study are available from the corresponding author, AM, upon reasonable request.

## References

[1] Musher DM, Thorner AR (2014). Community-Acquired Pneumonia. N Engl J Med.

[2] Walden AP, Clarke GM, McKechnie S, Hutton P, Gordon AC, Rello J (2014). Patients with community acquired pneumonia admitted to European intensive care units: an epidemiological survey of the GenOSept cohort. Crit Care.

[3] Jain S, Self WH, Wunderink RG, Fakhran S, Balk R, Bramley AM, et al. CDC EPIC Study Team. Community-Acquired Pneumonia Requiring Hospitalization among U.S. Adults. N Engl J Med. 2015;373(5):415–27. 10.1056/NEJMoa1500245. Epub 2015 Jul 14. 10.1056/NEJMoa1500245 PMC4728150 26172429

[4] Cunha BA (2006). The atypical pneumonias: clinical diagnosis and importance. Clin Microbiol Infect.

[5] Falcone M, Russo A, Tiseo G, Cesaretti M, Guarracino F, Menichetti F (2021). Predictors of intensive care unit admission in patients with Legionella pneumonia: role of the time to appropriate antibiotic therapy. Infection.

[6] Gacouin A, Revest M, Letheulle J, Fillatre P, Jouneau S, Piau C (2012). Distinctive features between community-acquired pneumonia (CAP) due to Chlamydophila psittaci and CAP due to Legionella pneumophila admitted to the intensive care unit (ICU). Eur J Clin Microbiol Infect Dis.

[7] Garnacho-Montero J, Garcia-Garmendia JL, Barrero-Almodovar A, Jimenez-Jimenez FJ, Perez-Paredes C, Ortiz-Leyba C (2003). Impact of adequate empirical antibiotic therapy on the outcome of patients admitted to the intensive care unit with sepsis. Crit Care Med.

[8] European Centre for Disease Prevention and Control (2019). Legionnaires’ disease - Annual Epidemiological Report for 2019.

[9] Metlay JP, Waterer GW, Long AC, Anzueto A, Brozek J, Crothers K (2019). Diagnosis and treatment of adults with community-acquired pneumonia. An official clinical practice guideline of the American Thoracic Society and Infectious Diseases Society of America. Am J Respir Crit Care Med..

[10] Woodhead M, Blasi F, Ewig S, Garau J, Huchon G, Ieven M (2011). Guidelines for the management of adult lower respiratory tract infections–full version. Clin Microbiol Infect.

[11] Valade S, Biard L, Lemiale V, Argaud L, Pène F, Papazian L (2018). Severe atypical pneumonia in critically ill patients: a retrospective multicenter study. Ann Intensive Care.

[12] Celli BR, MacNee W, ATS/ERS Task Force (2004). Standards for the diagnosis and treatment of patients with COPD: a summary of the ATS/ERS position paper. Eur Respir J..

[13] Chen DJ, Procop GW, Vogel S, Yen-Lieberman B, Richter SS (2015). Utility of PCR, Culture, and Antigen Detection Methods for Diagnosis of Legionellosis. In: Onderdonk AB, editor. J Clin Microbiol.

[14] Cunha BA, Burillo A, Bouza E (2016). Legionnaires’ disease. Lancet.

[15] Gramegna A, Sotgiu G, Di Pasquale M, et al. Atypical pathogens in hospitalized patients with community-acquired pneumonia: a worldwide perspective. BMC Infect Dis. 2018;18:677. 10.1186/s12879-018-3565-z PMC6299604 30563504

[16] Boer JW, Yzerman EPF. Diagnosis of Legionella infection in Legionnaires? disease. Eur J Clin Microbiol Infect Dis. 2004. Available from: http://link.springer.com/10.1007/s10096-004-1248-8. 10.1007/s10096-004-1248-8 15599647

[17] Helbig JH, Uldum SA, Bernander S, Luck PC, Wewalka G, Abraham B (2003). Clinical Utility of Urinary Antigen Detection for Diagnosis of Community-Acquired, Travel-Associated, and Nosocomial Legionnaires’ Disease.

[18] Yzerman EPF, den Boer JW, Lettinga KD, Schellekens J, Dankert J, Peeters M (2002). Sensitivity of three urinary antigen tests associated with clinical severity in a large outbreak of Legionnaires’ disease in The Netherlands. J Clin Microbiol.

[19] Zimmermann P, Curtis N (2019). The effect of antibiotics on the composition of the intestinal microbiota - a systematic review. J Infect.

[20] Rogers GB, Bruce KD, Martin ML, Burr LD, Serisier DJ (2014). The effect of long-term macrolide treatment on respiratory microbiota composition in non-cystic fibrosis bronchiectasis: an analysis from the randomised, double-blind, placebo-controlled BLESS trial. Lancet Respir Med.

[21] Munck C, Sheth RU, Cuaresma E, Weidler J, Stump SL, Zachariah P (2020). The effect of short-course antibiotics on the resistance profile of colonizing gut bacteria in the ICU: a prospective cohort study. Crit Care.

[22] Malhotra-Kumar S, Lammens C, Coenen S, Van Herck K, Goossens H (2007). Effect of azithromycin and clarithromycin therapy on pharyngeal carriage of macrolide-resistant streptococci in healthy volunteers: a randomised, double-blind, placebo-controlled study. Lancet.

