# Supplementary material for: Early discontinuation of combination antibiotic therapy in severe community-acquired pneumonia: a retrospective cohort study
Source: BMC Infect Dis. 2023 Sep 18;23:611. doi: 10.1186/s12879-023-08493-5 (PMC10506273; doi:10.1186/s12879-023-08493-5)
Supplement: Supplementary file 1 — Additional file 1: Supplementary Table 1. Diagnostic methods and treatment at admission of patients with atypical SCAP. [file 12879_2023_8493_MOESM1_ESM.docx]

**Supplementary Table 1** Diagnostic methods and treatment at admission of patients with atypical SCAP

|  | N (%) |
| --- | --- |
| Initial diagnostic method |  |
| *Legionella* UAT | 16 (89) |
| *Legionella* PCR and/or culture and/or serology | 1 (6) |
| *Mycoplasma pneumoniae* PCR | 1 (33) |
| *Chlamydia psittaci* PCR | 3 (60) |
| Diagnostic catch-up |  |
| *Legionella pneumophila* serogroup 1 | 1 (6) |
| *Mycoplasma pneumoniae* | 2 (66) |
| *Chlamydia psittaci* | 2 (40) |
| Initial treatment |  |
| Macrolide | 21 (81) |
| Fluoroquinolone | 6 (23) |
| Doxycycline | 1 (4) |
| De-escalation | 10 (38) |

Data are presented as number (%).
